# Highly indistinguishable photons from deterministic quantum-dot microlenses utilizing three-dimensional *in situ* electron-beam lithography

**DOI:** 10.1038/ncomms8662

**Published:** 2015-07-16

**Authors:** M. Gschrey, A. Thoma, P. Schnauber, M. Seifried, R. Schmidt, B. Wohlfeil, L. Krüger, J. -H. Schulze, T. Heindel, S. Burger, F. Schmidt, A. Strittmatter, S. Rodt, S. Reitzenstein

**Affiliations:** 1 Institut für Festkörperphysik, Technische Universität Berlin, Hardenbergstraße 36, Berlin D-10623, Germany.; 2 Zuse-Institut Berlin (ZIB), Takustraße 7, Berlin D-14195, Germany.

## Abstract

The success of advanced quantum communication relies crucially on non-classical light sources emitting single indistinguishable photons at high flux rates and purity. We report on deterministically fabricated microlenses with single quantum dots inside which fulfil these requirements in a flexible and robust quantum device approach. In our concept we combine cathodoluminescence spectroscopy with advanced *in situ* three-dimensional electron-beam lithography at cryogenic temperatures to pattern monolithic microlenses precisely aligned to pre-selected single quantum dots above a distributed Bragg reflector. We demonstrate that the resulting deterministic quantum-dot microlenses enhance the photon-extraction efficiency to (23±3)%. Furthermore we prove that such microlenses assure close to pure emission of triggered single photons with a high degree of photon indistinguishability up to (80±7)% at saturation. As a unique feature, both single-photon purity and photon indistinguishability are preserved at high excitation power and pulsed excitation, even above saturation of the quantum emitter.

## Abstract (web summary)

Single indistinguishable photon sources with high flux rates and purity are needed in quantum communications. Here, Gschrey *et al*. use three-dimensional electron-beam lithography to pattern deterministic quantum-dot microlenses and demonstrate enhanced photon-extraction efficiency and photon indistinguishability.

## Graphical abstract

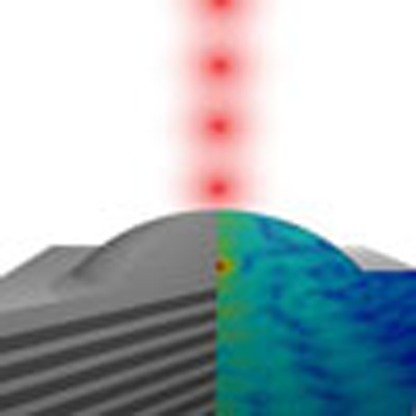

The prospect of realizing building blocks for long-distance quantum communication is a major driving force for the development of advanced nanophotonic devices^1^. Since the first demonstration of optically^2^ and electrically^3^ driven quantum-dot-based single-photon sources, significant progress has been achieved in this field with respect to the fabrication of efficient sources^4^,^5^,^6^,^7^,^8^,^9^,^10^, enabling even quantum key distribution^11^ using electrically driven devices. More recently, even spin-photon entanglement^12^,^13^ and quantum teleportation^14^,^15^ have been demonstrated in semiconductor systems. These results are considered as crucial steps towards the realization of a quantum repeater network. The related work has almost exclusively been performed on self-assembled quantum dots (QDs) in combination with conventional device technology, where spatial and spectral matching is random, resulting in a small yield of usable devices. At this point, and in particular with quantum device networks in mind, it is clear that further progress in this field towards real applications will crucially rely on deterministic device technologies^16^, such as *in situ* lithography techniques as introduced in ref. 17. These technologies will for instance enable the fabrication of bright quantum-light sources with precisely controlled emission energies, which is a key requirement for, e.g., entanglement distribution via Bell-state measurements.

Besides the above mentioned practical aspects, light sources for quantum communication have to combine high photon-extraction efficiency (PEE), high flux rate, high suppression of multi-photon emission events and a high degree of photon indistinguishability. Using semiconductor-QD-based approaches, great progress has been achieved on individual aspects, such as PEEs up to 70–80% (refs 6, 8) or close-to-ideal photon indistinguishability^18^. So far these features have not been realized within a single device. For instance, microcavity-based single-photon sources typically suffer from an enhanced probability of multi-photon emission events at high pump rates because of an intensified contribution of uncorrelated background emission^5^,^7^,^19^. This results in a trade-off between low *g*^(2)^(0)-values and high photon-flux rates. Moreover, resonator structures provide narrow-band enhancement of QD emission which leads to limitations regarding the generation of polarization-entangled photon pairs^20^. Photonic nanowires^6^, on the other hand, allow for low *g*^(2)^(0)-values even at high single-photon emission rates, but cannot easily be integrated into electrically driven photonic circuits and are likely to suffer from fluctuations of surface charges which seems to be an open issue regarding the emission of indistinguishable photons^21^.

Here we report on a novel approach that opens the way to combine all required features in a single device concept. It is based on deterministically fabricated single-QD microlenses with an integrated mirror section at the bottom. QDs with selected emission properties can be chosen to match the envisaged applications. As we will show, these quantum-light sources can be realized with a very flexible and robust fabrication technique and allow for broadband-enhanced single-photon emission with very-high photon-flux rates in combination with vanishing *g*^(2)^(0)-values and a high degree of photon indistinguishability even at saturation of the active QD. Broadband enhancement of the PEE is of particular interest for boosting the generation of polarization-entangled photon pairs from the biexciton–exciton cascade of QDs with diminishing fine-structure splitting^22^. Another appealing aspect of the microlens approach is the possible application in a straightforward way in various material systems such as wide-bandgap II/VI-semiconductors or InGaAs QDs on (111)-oriented GaAs with fine-structure splitting close to zero^23^,^24^,^25^ for which the growth of cavity structures is still challenging or has not been mastered yet at all. In addition to the strongly enhanced extraction efficiency, our microlens approach comes along with the benefit that an exciting laser is also focused onto the QD which allows for a reduced laser power. This is of special interest for resonance fluorescence spectroscopy which will benefit from an increased signal-to-noise ratio because of an enhanced PEE and reduced stray light from the exciting laser.

## Results

### Lens fabrication

**Figure 1:**
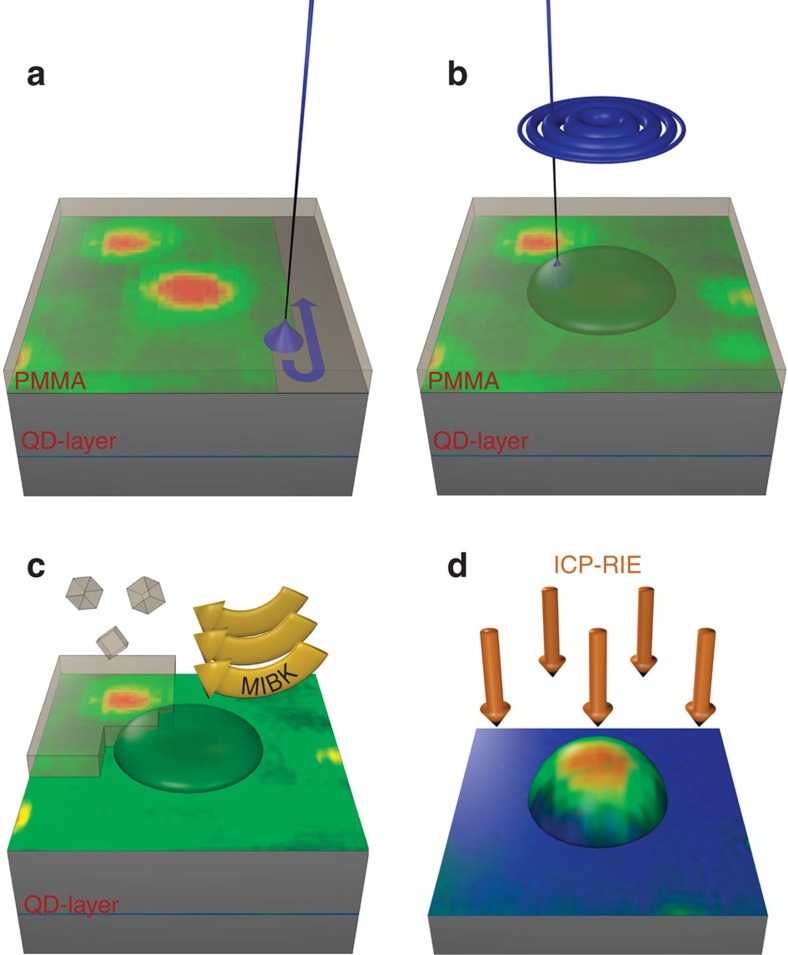
The lens fabrication process. (**a**) The sample's luminescence is mapped by cathodoluminescence spectroscopy. Along this, the resist is exposed to an electron dose around 10 mC cm^−2^ and becomes soluble upon development. (**b**) On top of suitable QDs, lens structures are written into the resist by cross-linking the afore cracked PMMA chains by using an additional electron dose. The lens shape is defined by writing concentric circles into the resist and by carefully adjusting the respective electron doses. (**c**) Singly exposed resist is removed by applying the solvent methylisobutylketon and the lens shape forms in the inverted regions. (**d**) Upon dry etching the lens profile is transferred from the inverted PMMA into the semiconductor. The bottom DBR section is omitted for a better display format.

**Figure 2:**
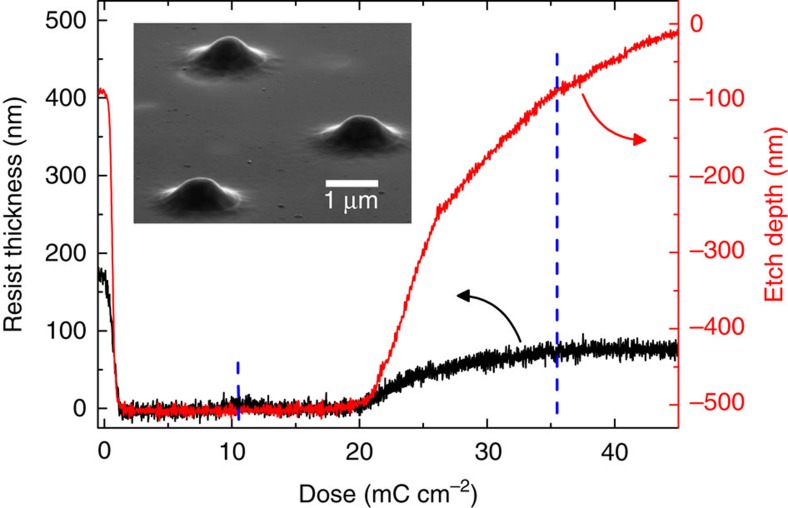
Contrast curve and etch profile. Prerequisite for etching lens structures is to create 3D lens profiles in the resist. Left axis, black curve: height profile of 190 nm PMMA after development as a function of applied electron dose. Between 0 and 20 mC cm^−2^ we observe the characteristics of a positive tone resist. Then cross-linking starts and the remaining height is directly related to the applied dose. Right axis, red curve: Etch depth in GaAs that corresponds directly to the resist thickness as given by the black curve. The resist profile is precisely transferred into the semiconductor as proven by scanning electron micrographs of etched lenses (inset) that resemble targeted Gaussian profiles.

We apply our deterministic quantum device concept by integrating pre-selected single QDs into monolithic microlenses using *in situ* three-dimensional (3D) electron-beam lithography in a low-temperature cathodoluminescence (CL) system^26^. Although our microlens approach can be adapted to all semiconductor materials with embedded quantum emitters, we have chosen the technically mature InGaAs/GaAs material system for demonstration purposes. Moreover, numerical calculations demonstrate that a future mirror design can highly improve the PEE to values above 80% (see Supplementary Fig. 1). The generic lens fabrication process is sketched in Fig. 1a–d. After spin-coating the sample with the electron-beam sensitive resist polymethyl methacrylate (PMMA)^27^ the QD luminescence is characterized via CL spectroscopy mapping at a cryogenic temperature of 5 K (a). Within this first step, the positive tone resist is already exposed and converted to a soluble layer. In the second processing step (b), which is performed directly afterwards also at cryogenic temperatures, the microlenses are structured by means of 3D electron-beam lithography and local overexposure of the resist. This is done by writing concentric circles with carefully adjusted electron doses, centred at the positions of the pre-selected QDs. After development at room temperature (c), the overexposed resist remains as an etch mask on the sample which is transferred into the semiconductor material in the subsequent plasma-etching step (d). Here, the exposure dose profile determines the local thickness of the PMMA-etch mask which allows us to precisely tailor the microlens shape in 3D (Fig. 2). The etch depth was carefully chosen to remove the surrounding QDs while obtaining the lens with the single QD therein. A scanning electron microscopy image of readily processed microlenses with a base width of 1.5 μm is presented in the inset of Fig. 2, demonstrating that microlenses with a given shape can precisely be realized. This result clearly proves that our low-temperature electron-beam lithography approach allows us to align 3D nanostructures with an arbitrary shape to pre-selected QDs, featuring an overall alignment accuracy of 34 nm as reported in ref. 28. The necessity of such a high alignment accuracy is evident from the calculations presented in Supplementary Fig. 2: a lateral displacement of the lens of 100 nm easily results in a drop of the PEE of 5%.

### Lens design

**Figure 3:**
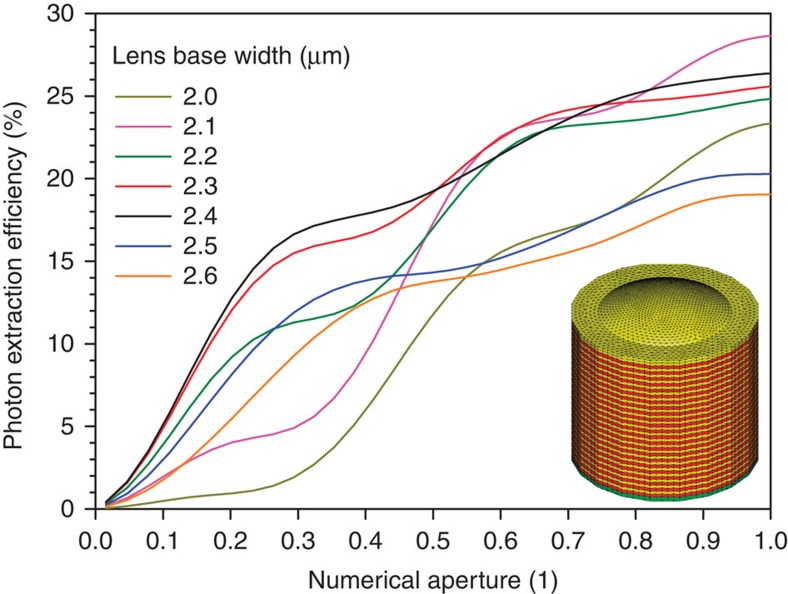
Numerical results. Numerical results for the PEE for a hemispherical section-shaped lens and a bottom-DBR as a function of the NA of the light-collection optics. The diverse curves correspond to different base widths of the lenses with a common height of 400 nm. The inset displays the meshed model structure as used for the calculation (yellow: GaAs, red: AlGaAs).

The determination of optimal lens parameters is based on solving Maxwell's equations with a state-of-the-art finite-element solver^29^. The model structure consists of a lower distributed Bragg reflector (DBR) section with 23 pairs of Al_0.9_Ga_0.1_As/GaAs, a 65-nm GaAs spacer, a point source and the lens structure on top. Lenses of Gaussian-, Weierstrass- and hemispheric-type^30^ were modelled to maximize the PEE. Best results were obtained for shallow hemispheric sections with heights of 400 nm and base widths ∼2.4 μm. Our lens design is an optimization of the trade-off between direct outcoupling and outcoupling of the back-reflected light. Fig. 3 displays calculated PEE values for lenses of different base-diameters as a function of the numerical aperture (NA) of the light-collection optics. The inset shows the model structure. For the experimental studies presented in the following, lenses with a base width of 2.4 μm were processed as advised by the calculations where a PEE of 18% is predicted for a NA of 0.4. The highly directed emission and optimal lens effect for that geometry is also expressed by the angular emission profile as presented in Supplementary Fig. 3. The lens with the diameter of 2.4 μm gains the highest amplitude of directed emission. Supplementary Fig. 4 shows the corresponding field distribution in the *x*–*z* section plane for the 2.4 μm lens. Again, the focusing effect can be clearly seen.

### Optical characterization

**Figure 4:**
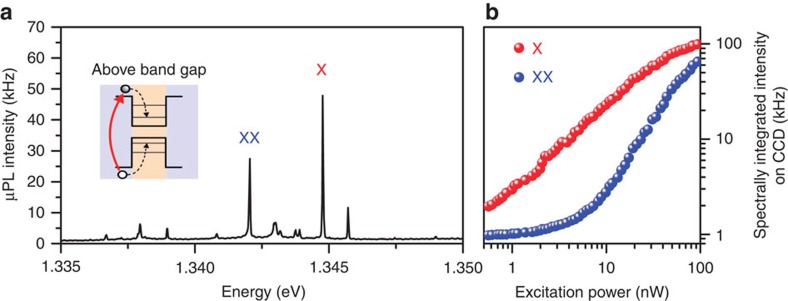
*μ*PL characterization of a microlens-boosted QD. (**a**) Emission lines from the single QD that stem from the exciton's (X) and the biexciton's (XX) recombination, measured at a temperature of 6 K. (**b**) Spectrally integrated intensity on the charge-coupled device camera for X and XX emission versus excitation power. In saturation a PEE of (23±3)% can be derived for the emission of the QD.

We demonstrate the high optical performance of single-QD microlenses with a bottom DBR and their potential to act as non-classical light sources in the field of quantum communication via quantum-optical studies at low temperature. Fig. 4a presents a *μ*PL spectrum of such a microstructure excited non-resonantly at a wavelength of 810 nm using a picosecond-pulsed Ti:sapphire laser at a repetition frequency of 80 MHz. A microscope objective with NA of 0.4 was used to collect the luminescence from the QD. The labelled emission lines correspond to the neutral exciton (X) and the biexciton (XX) states of a single QD integrated into the microlens, where the assignment was deduced from power- and polarization-dependent measurements (not shown). The spectrally integrated intensity of the X and XX emissions is depicted in Fig. 4b as a function of the excitation power. In saturation we observe very-high count rates of >100 kHz for the X-line. The excitation-power-dependent emission from the excitonic lines allows us to determine a PEE as high as (23±3)% (see Methods for more details), which is in very good agreement with the simulations presented in Fig. 3. We would like to note that the numerical model uses a point source and an idealized 3D geometry. The deviation of these from the real experimental conditions can explain the slight difference between experimentally (23±3)% and numerically (18%) obtained PEEs.

**Figure 5:**
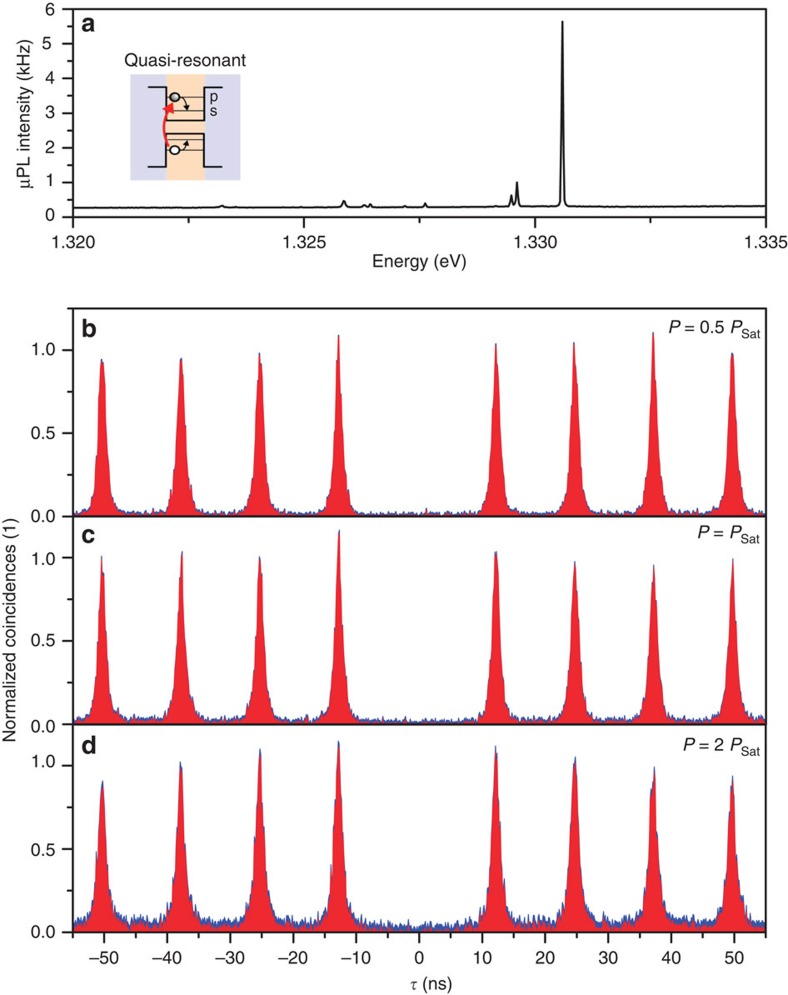
Single-photon emission. (**a**) *μ*PL spectrum of the quantum dot under quasi-resonant excitation at low excitation power. (**b**–**d**) *g*^(2)^(*τ*) results for the X^+^ emission at pump powers of 0.5·*P*_Sat_, *P*_Sat_ and 2·*P*_Sat_. Analysis of corrected data (red) yields a *g*^(2)^(0)<0.01 independent of the optical excitation power.

Next, the quantum nature of the emission of a deterministic single-QD microlens was studied via excitation-power-dependent photon autocorrelation measurements under pulsed quasi-resonant excitation (Fig. 5a) by using a fibre-coupled Hanbury-Brown and Twiss (HBT) setup. Fig. 5b–d displays the corresponding *g*^(2)^(*τ*) histograms of the positively charged exciton state X^+^ for an excitation power of *P*=0.5·*P*_Sat_, *P*_Sat_ and 2·*P*_Sat_, where *P*_Sat_ denotes the excitation power at which the emission line saturates. We observe almost excitation-power-independent values of *g*^(2)^(0)≤0.01 for up to two times the saturation power. This unique feature of the deterministic microlenses proves that our structures can act as bright single-photon sources with close-to-ideal quantum properties even at maximum photon flux. Although the excitation power independence of *g*^(2)^(0) has for example also been observed in photonic nanowires^6^, we go a significant step further by demonstrating that our structures not only show a close-to-ideal single-photon emission but also at the same time a high degree of photon indistinguishability. The latter is of crucial importance for advanced quantum communication concepts such as the quantum repeater, which relies on entanglement distribution via Bell-state measurements^31^,^32^,^33^.

**Figure 6:**
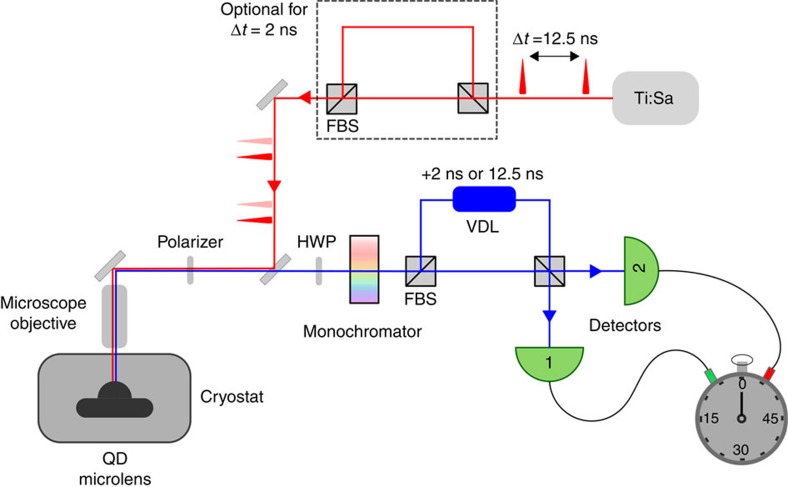
Hong–Ou–Mandel setup. The QD microlens is excited via a pulsed picosecond Ti:Sapphire laser (*f*=80 MHz) with a pulse separation of 12.5 ns. A Mach–Zehnder interferometer in the excitation path enables us to realize a two-pulse excitation scheme, where consecutive pulses are separated by 2 ns. The quantum-dot emission was then collected with a microscope objective and spectrally filtered by a grating monochromator. The two-photon interference experiments can be carried out using an asymmetric fibre-based interferometer with a path-length difference of 12.5 and 2 ns, respectively. Second-order correlation histograms were recorded with two silicon APDs connected to the output ports of the second FBS. For measurements in cross-polarized configuration a half-wave plate (HWP) is introduced in one interferometer arm. FBS, fibre-coupled beamsplitter; VDL, fibre-based variable optical delay.

**Figure 7:**
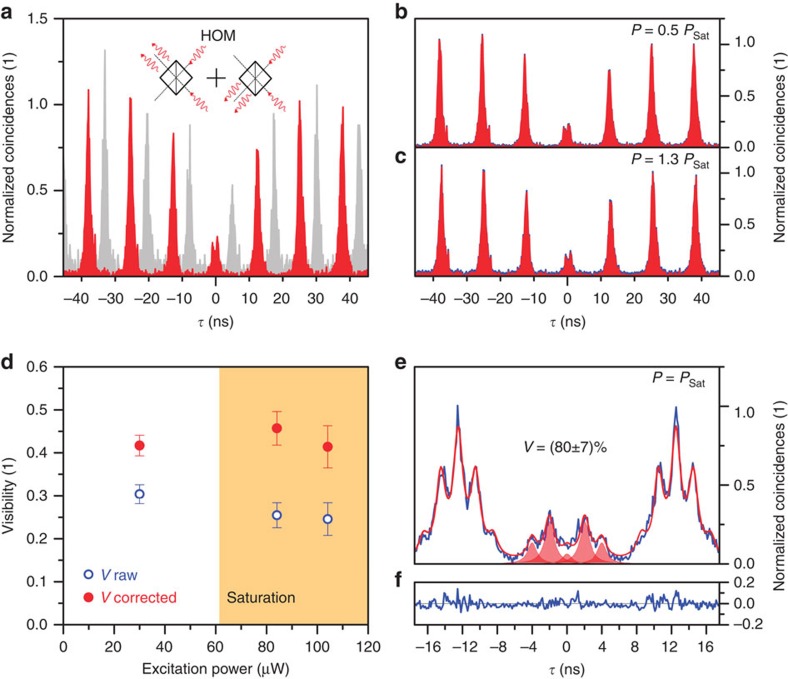
Two-photon interference. (**a**) *g*^(2)^(*τ*) measured with a HOM-type setup for co- (red) and cross- (grey) polarized photons interfering at the second beamsplitter (data for cross-polarized configuration have been shifted in time for clarity). (**b**,**c**) HOM histograms for co-polarized photons and increasing optical pump powers of 0.5·*P*_Sat_ and 1.3·*P*_Sat_. (**d**) Two-photon interference visibility in dependence on the excitation power at an excitation pulse separation of 12.5 ns. The shaded region indicates the saturation of the QD state. (**e**) HOM histogram obtained for excitation pulses separated by 2 ns at saturation pump power. The fit corresponds to Lorentzian peaks and we extract a corrected (raw) two-photon interference visibility of (80±7)% ((76±4)%) from the data. (**f**) Residual of fit to the data shown in (**e**).

To investigate the indistinguishability of photons emitted from deterministic single-QD microlenses, we use a fibre-coupled Hong–Ou–Mandel (HOM)-type setup as displayed in Fig. 6. Here, consecutively emitted single photons from the QD interfere on the second beamsplitter when entering from the two different input ports. A half-wave plate (HWP) allows for switching the polarization of photons in one arm of the interferometer with respect to the other arm to make them clearly distinguishable. In case of ideal indistinguishability, both photons coalesce in a two-photon Fock state. This is reflected in the measured second-order correlation histogram *g*_HOM_^(2)^(*τ*) since coincidences at a time delay *τ*=0 become absent. The central peak vanishes and the corresponding visibility of two-photon interference (TPI)—essentially characterizing the mean photon-wavefunction overlap—approaches unity. To investigate the indistinguishability of photons emitted from the QD microlens, we performed excitation power-dependent measurements of *g*_HOM_^(2)^(*τ*) on the X^+^ emission line of the QD, whose spectrum under quasi-resonant excitation is shown in Fig. 5a. The resulting *g*_HOM_^(2)^(*τ*) histograms for an excitation-pulse separation of *Δt*=12.5ns are shown in Fig. 7a–c, where the blue curve corresponds to the measured raw data and the red curve to background-corrected data (see Methods).

The extracted values of *g*_HOM_^(2)^(0) again remain almost constant with increasing excitation power even beyond saturation. A behaviour that was already observed for the bare *g*^(2)^(0) value (Fig. 5). For the quantitative analysis we take into account the finite probability of multi-photon emission events and the imperfections of the HOM setup, *R*/*T*=0.51/0.49 and 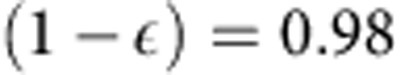, where *R* and *T* denote the reflection and transmission coefficients of the fibre-based beamsplitter and 
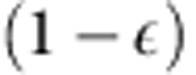
 is the maximal interference fringe contrast obtained for the balanced interferometer, similar to ref. 21. A detailed analysis of the data is presented in Fig. 7e and reveals the TPI visibility. Well above saturation a corrected (raw) value of *g*_HOM_^(2)^(0)=0.271±0.019 (0.373±0.015) is extracted, revealing a corrected (raw) TPI visibility of *V*_HOM_=(46±4)% ((26±3)%). One possible explanation for the remaining discrepancy to an ideal source of indistinguishable photons with *V*=1 is the presence of spectral diffusion on a timescale comparable to the excitation pulse separation (12.5 ns) (ref. 21). This might lead to a reduced spectral overlap at the HOM beamsplitter, thus limiting the TPI visibility. To test this interpretation, we modified the TPI setup and applied a two-pulse excitation scheme with a pulse separation of 2.0 ns (ref. 21)—a scheme which is commonly used to achieve optimal TPI visibilities.

The corresponding histogram measured at saturation of the X^+^-state can be seen in Fig. 7d. In contrast to the previous TPI histograms the central coincidence maximum reveals a five peak structure with an intensity ratio of 1:2:0:2:1 for perfect indistinguishable photons. A fit to the data assuming Lorentzian profiles with integrated intensity ratios according to the theoretical prediction (residual can be seen in Fig. 7f), reveals a corrected (raw) TPI visibility of *V*_HOM_=(80±7)% ((76±4)%). This remarkable increase of almost a factor of two in TPI as compared to a pulse separation of 12.5 ns proves that inhomogeneous broadening due to spectral diffusion of the emission line was limiting the TPI to a certain degree but can be decreased by reducing the excitation pulse separation. Moreover, to achieve interference visibilities close to unity, pure dephasing processes, causing homogeneous broadening, have to be eliminated. The linewidth of photons emitted from the source is required to be Fourier-transform limited according to the relation *T*_2_=2*T*_1_, where *T*_2_ denotes the coherence time and *T*_1_ the spontaneous emission lifetime of the emitter. To further increase the TPI visibility, cavity-integrated microlenses^34^,^35^ excited via a strict resonant excitation scheme, reducing the exciton lifetime and improving the coherence properties, are promising candidates.

## Discussion

The above results demonstrate the superior capability of deterministically integrated QD-microlenses for the generation of single indistinguishable photons on demand, featuring high suppression of multi-photon emission events at high flux rates. An important feature that can be directly related to the fact that parasitic uncorrelated background emission is almost completely eliminated in the used technology approach which makes it indispensable for future applications in the field of optical quantum information technologies. The photons emitted by the QD showed no significant degradation in their indistinguishability even at pump powers beyond saturation. Additionally, numerical calculations (see Supplementary Fig. 1) predict that next-generation microlens structures might feature PEEs of above 80% (for large-NA applications), by introducing a gold mirror at the bottom instead of a DBR. Such a PEE is very comparable to maximum values obtained for the photonic wire approach and for microcavity-based single-photon sources. Furthermore, the gold mirror can directly be utilized as backside contact for charge carrier injection or strain-tuning^36^. We would like to stress that this still goes along with the unique possibility of combining lowest *g*^(2)^(0)-values, a high single-photon flux, and a high degree of indistinguishability in deterministic quantum light sources, paving the way towards the realization of a quantum repeater within an integrated semiconductor technology platform.

In summary, we have demonstrated a versatile and powerful method to enhance the PEE from semiconductor nanostructures by fabricating deterministic microlenses. The investigated structures have proven that our approach has a high potential to boost real applications of quantum light sources. Because of their broadband enhancement of the PEE and the straightforward fabrication process the deterministic microlenses will be of particular interest for the realization of polarization-entangled photon pairs and sources of indistinguishable photons in quantum repeater networks. In combination with our numerical results, we pave the way towards the fabrication of such sources with an overall (creation and collection) efficiency of 80%.

## Methods

### Sample growth and structure

The sample was grown by metal–organic chemical vapour deposition on GaAs(001) substrate. First, 300 nm of GaAs were deposited, followed by a DBR consisting of 23 pairs of *λ*/4-layers of 77 nm Al_0.9_Ga_0.1_As and 65.7 nm GaAs. Next, 65 nm of GaAs was grown, followed by self-organized InGaAs QDs in the Stranski–Krastanow growth mode at a temperature of 500 ^°^C during a growth interruption of 35 s. Finally, the QDs were capped by 400 nm of GaAs. The GaAs capping layer provides the material for the final lens structures after etching. The central wavelength for the DBR is *λ*=935 nm.

### Cathodoluminescence lithography setup

Samples are mounted on the cold finger of a He-flow cryostat (5–300 K) in a JEOL JSM-840 scanning electron microscope upgraded with a custom-made lithography system. The samples' luminescence is excited by the electron beam of the microscope with an acceleration voltage of 10 kV and a beam current of 0.5 nA and focused into a 0.5-m spectrometer by an elliptical mirror. The spectrometer was equipped with a 600 grooves per mm grating for the presented cathodoluminescence lithography (CLL) experiments and the luminescence is detected by a liquid-nitrogen (LN)-cooled Si-charge-coupled device camera.

### Electron-beam lithography and sample processing

The deterministic microlenses are fabricated in the following way. First, we spin-coat a 225-nm thick layer of PMMA on the sample before recording CL intensity maps in the CL-system at a temperature of 5 K and a dose of around 10 mC cm^−2^, while great care is taken to achieve a homogeneous distribution of the applied dose. Out of the number of embedded QDs we select target QDs which show reasonably spatially isolated emission spots and matching emission characteristics. In the subsequent *in situ* electron-beam lithography step we pattern lens structures into the resist. We therefore write concentric circles which are centred at the position of a target QD under variation of the dose (highest dose at the centre and lower doses towards the edge of the lens) to form the correct dose profile as sketched in Fig. 1b. The actual electron dose for every written circle is derived from the contrast and etch-resistance curves of the resist (Fig. 2). We would like to point out that the whole selection and *in situ* lithography process of typically 10 deterministic QD-microlenses in a write-field of 23.9 μm by 17.9 μm is performed within 4 min without moving the sample. Afterwards, the sample is transferred out of the CL-system and is developed in a mixture of methylisobutylketon and isopropyl alcohol at room temperature. Next, dry etching is performed by inductively coupled-plasma reactive-ion etching under a pressure of 0.08 Pa with 100 W ICP coil power and −213 V substrate bias voltage. A combination of Cl_2_:BCl_3_:Ar, with a ratio of 1.3:4.3:1.1, is used to reach a selectivity of GaAs against unexposed PMMA of about two. Realizing an etch depth of up to 430 nm, the lens profile was transferred from the inverted PMMA into the semiconductor with a simultaneous removal of the QD layer around each lens.

### Determination of the PEE

To determine the PEE we excited the QD with a pulsed Ti:Sapphire laser (*f*=80 MHz) at saturation of the X emission line (Fig. 4), collected the luminescence using a microscope objective with a NA of 0.4 and spectrally filtered the X emission using the monochromator. Under these conditions we observed a total count-rate of 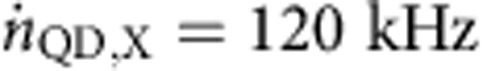 at the avalanche photo-diodes (APDs) coupled to the beamsplitter of the Hanbury-Brown and Twiss setup. For the single-photon flux into the first lens the setup efficiency was derived using a tunable laser which was focused onto a gold mirror mounted in the cryostat and tuned to the wavelength of the X emission line. The laser was attenuated using neutral density filters in front of the monochromator to achieve APD count-rates comparable to those observed for the QD emission. Taking into account the laser power, the reflection of the gold mirror, the transmission of the cryostat window, the attenuation of the density filters, and the maximal count-rates on the APDs we determined a setup efficiency of *η*_Setup_=0.8%. From the detected count-rate 
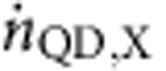
, the setup efficiency *η*_Setup_ and the laser repetition rate *f*, we finally deduce a PEE of 
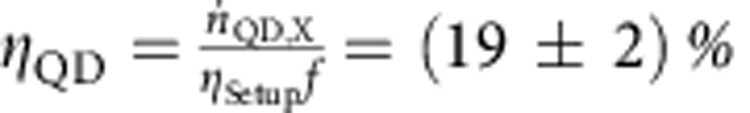
 for the X emission. Considering that only one excitonic complex can radiatively decay at a time we sum up the contributions of all excitonic lines to achieve *n*_QD_=(23±3)%.

### Quantum-optical setup

Figure 6 shows a schematic of the setup. The sample is mounted in a Helium-flow cryostat and cooled down to temperatures between 5 and 15 K. Excitation was performed via a pulsed picosecond Ti:sapphire laser at 810 and 915 nm, respectively. The HWP was used to rotate the polarization of QD photons parallel to the optical table while the polarizer allows for suppression of the laser background. After high-resolution spectral filtering of the emission lines, quantum optical studies can be carried out using fibre-coupled 50:50 beamsplitters and single-photon detectors. For details of the HBT setup please refer to ref. 26^26^. The HOM-type TPI experiments were carried out using an asymmetric Mach–Zehnder interferometer (Δ*t*=12.5 ns and Δ*t*=2 ns, respectively) based on polarization maintaining fibres and two fibre-coupled beamsplitters. A HWP is used to switch the polarization in one arm of the interferometer^37^,^38^. To control the temporal (and spatial) overlap of the photon wavepackets at the second beamsplitter, a fibre-based variable optical delay is introduced in one interferometer arm, enabling a fine-tuning of the photon arrival time with 3 ps precision.

### Numerical method

The calculation of the PEE was performed in the framework of a finite-element method by using the commercially available software-package *JCMsuite* by JCMwave. A full-3D model structure with a lens on top and a mirror at the bottom was created and the structures' parameters were varied as described in the text. The resulting light intensity distribution from a point source acting as the buried QD was calculated in the framework of full-3D light-scattering. The angular integration of intensity for a given NA was performed by evaluating the far-field distribution. For an example see Supplementary Fig. 4 where an electric field distribution in the *x*–*z* section plane is displayed.

### Background correction in correlation measurements

For the corrections applied to the raw data we took into account coincidences arising from the detection of photons from the QD emission and the laser background or APD dark counts, respectively. The laser background was evaluated from a comparison of the APD count-rates close to the emission line for above-band excitation with the count-rates for quasi-resonant excitation. By using this method we evaluate the contribution from the laser background to be 250–750 Hz for the Hanbury-Brown and Twiss correlation measurements and 75–180 Hz for the TPI correlation measurements. Together with an APD dark count-rate of 50 Hz the entire uncorrelated coincidences per time-bin are given by 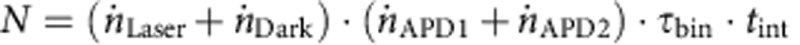 with 
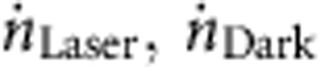
 being the laser background rate and dark count-rate, 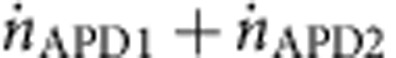 the count-rates on the two APDs, *τ*_bin_ the time-bin width, and *t*_int_ the overall integration time.

## Additional information

**How to cite this article:** Gschrey, M. *et al*. Highly indistinguishable photons from deterministic quantum-dot microlenses utilizing three-dimensional *in situ* electron-beam lithography. *Nat. Commun.* 6:7662 doi: 10.1038/ncomms8662 (2015).

## Supplementary Material

Supplementary Information Supplementary Figures 1-4
