# Supplementary material for: Highly indistinguishable photons from deterministic quantum-dot microlenses utilizing three-dimensional in situ electron-beam lithography
Source: Nat Commun. 2015 Jul 16;6:7662. doi: 10.1038/ncomms8662 (PMC4518279; doi:10.1038/ncomms8662)
Supplement: Supplementary Information — Supplementary Figures 1-4 [file ncomms8662-s1.pdf]

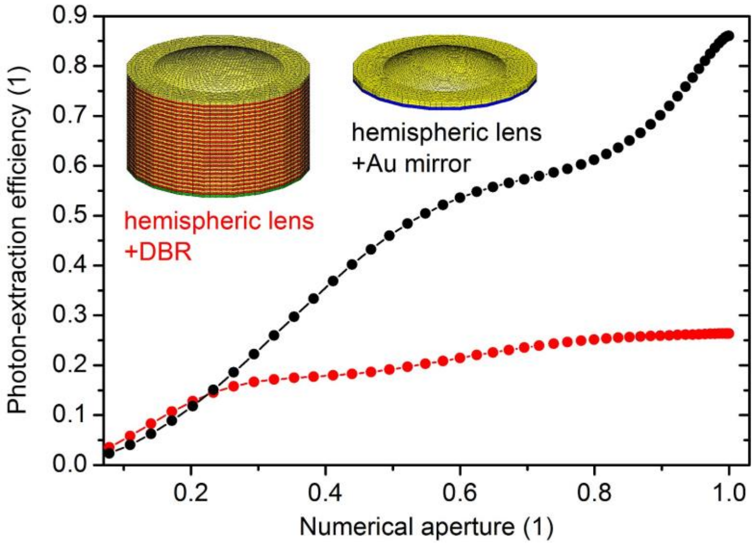

Supplementary figure 1 | Comparison of the photon-extraction efficiency for different microlens-mirror structures. To assess the impact of different backside mirrors on the photon-extraction efficiency (PEE) for microlens-mirror structures, two different mirror approaches are numerically compared. The first one comprises a DBR as described in the main text and the second one is based on a gold layer below the quantum dot (QD) acting as mirror. In both cases an optimized lens shape with respect to the given mirror is included in the model structure. Black: Hemispheric-section lens (height = 400 nm, base width = 2.0 µm) with a gold mirror 150 nm below the QD. Red: Hemispheric-section lens (height = 400 nm, base width = 2.4 µm) with a DBR 65 nm below the QD as described in the main text. The x-axis gives the numerical aperture of the external light-collection optics. The insets show the respective model structures (yellow = GaAs, red = AlGaAs, blue = Au). All data is calculated by using the finite-element solver JCMsuite in full-3D light-scattering mode as described in the main text. The numerical results clearly demonstrate that the Au-mirror design is superior for larger numerical apertures (NAs) of the external light-collection optics. This is mainly a result of the finite acceptance angle of the DBR of approx. 19°. For larger angles of downward QD emission, the reflectivity of the DBR decreases drastically and the light is no longer reflected back. In contrast, the reflectivity of the Au-mirror is almost independent of the angle of incidence and the PEE of the microlens-Au structure reaches even 86 % which is at the top of state-of-the-art results (see main text). However, we would like to point out that in the range of smaller NAs (up to about 0.22) that is technological relevant for fiber-coupling the microlens-DBR structure performs even better than the lens in combination with an Au mirror.

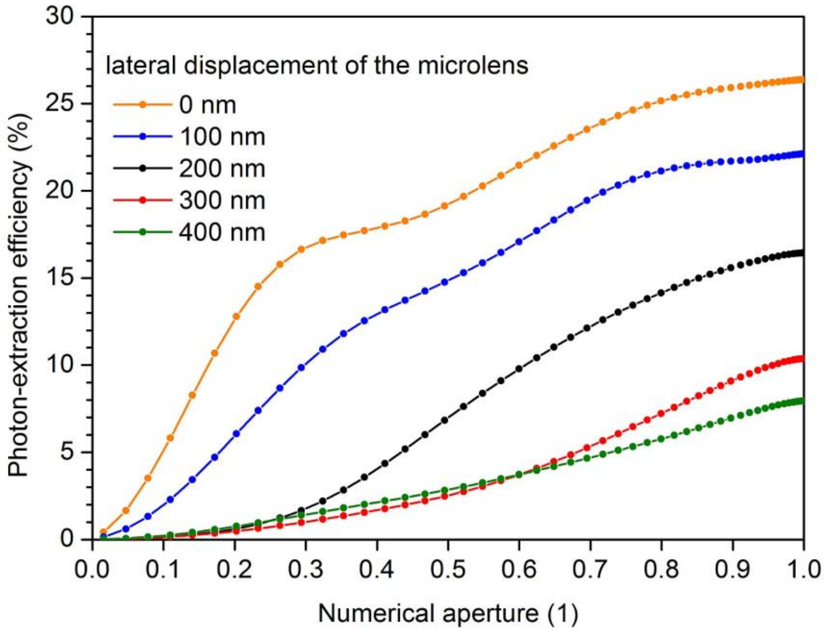

Supplemental figure 2 | Dependence of the photon-extraction efficiency on the lateral displacement of the microlens. To evaluate the impact of a possible lateral displacement of the lens as a consequence of a non-ideal fabrication process, numerical modeling is performed for the 2.4 µm microlens-DBR structure from the main text. Five different lateral displacements ranging from 0 to 400 nm are evaluated. The x-axis gives the numerical aperture of the external light-collection optics. A lateral displacement of 100 nm already results in a drop of PEE from 18 % to 13 % for, e.g., a NA 0.4. This highlights the importance of a deterministic fabrication process with a high lateral accuracy like our CLL technique for which we determined a lateral processing accuracy (standard deviation) of 34 nm for the full process.

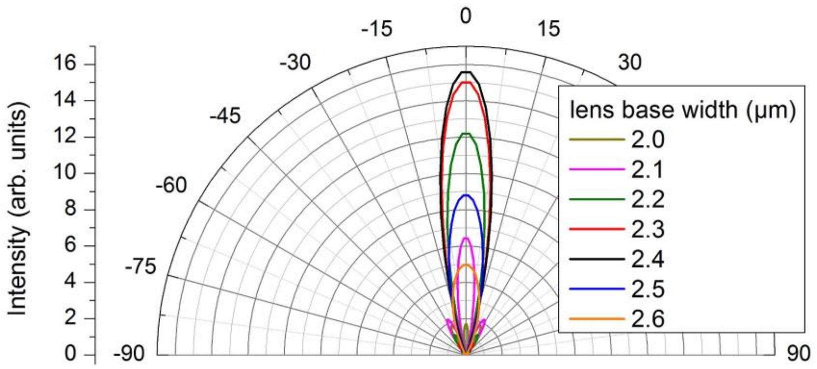

Supplemental figure 3 | Angular emission profiles for the hemispheric-section lenses with a bottom DBR. Calculated emission profiles for the hemispheric-sections lenses from Fig. 3 in the main text. All lenses have a height of 400 nm and a base width as indicated in the figure. All lens structures show pronounced directional emission with the highest amplitude for the 2.4 µm lens.

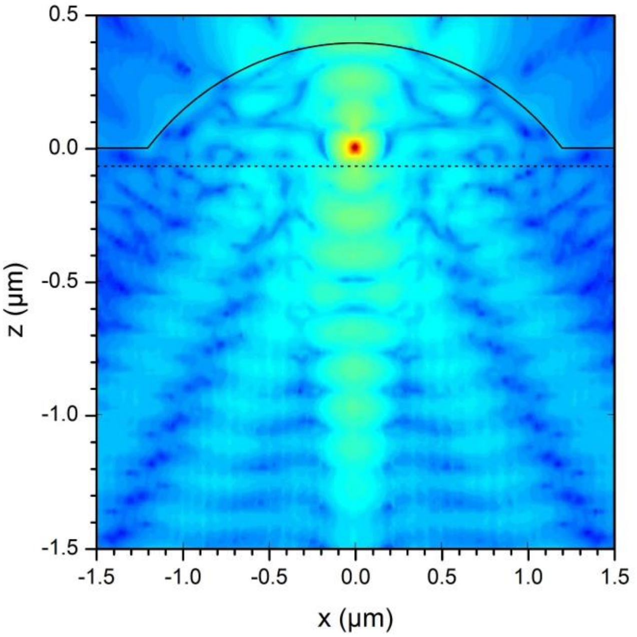

Supplemental figure 4 | Electric field distribution in the near-field regime for the 2.4 µm hemispheric-section lens with a bottom DBR. Calculated near-field distribution for the hemispheric-section lens with a height of 400 nm and a base width of 2.4 µm. The semiconductor-air interface is marked by the solid black line. Below the point source (bright red spot), distinct field modulations due to the bottom DBR (starting at the dashed black line) are visible (the bottom DBR is truncated in the figure for a better display format).
